# Reconstruction of a Central Full-Thickness Glenoid Defect Using Osteochondral Autograft Technique from the Ipsilateral Knee

**DOI:** 10.1007/s43465-020-00190-8

**Published:** 2020-08-30

**Authors:** Marius Junker, Jörn Kircher

**Affiliations:** 1 Orthopedic University Hospital Friedrichsheim, Marienburgstraße 2, 60528 Frankfurt, Germany; 2 Department of Shoulder and Elbow Surgery, ATOS Klinik Fleetinsel Hamburg, Hamburg, Germany

**Keywords:** Shoulder, Glenoid, Defect, Autograft

## Abstract

**Background:**

Osteochondral defects (OCDs) of the shoulder represent a typical clinical problem and are difficult to manage. OCDs of the upper extremity are less common than those of the lower extremity. The incidence is reported to be between 5–17% in which the humerus is affected more frequently than the glenoid. OCD is often accompanied with symptoms and may appear secondary to trauma, instability or prior operation. The problem of the lesions is the missing blood circulation which makes the healing impossible. The hazard of OCDs is the progression to osteoarthritis. In spite of the effectiveness of total shoulder arthroplasty it is not the first option for young and active patients. The therapy options of OCD depend on the size and localization of the defect.

**Purpose:**

The aim of this multimedia article is to reveal a therapy option for OCDs of the glenoid.

**Methods:**

In this case we present the reconstruction of a central full-thickness osteochondral glenoid defect with an osteochondral autograft from the ipsilateral knee which was withdrawn using the OATS-Technique (Arthrex, Naples, Florida) to address the chondral as well as the osseous pathology. To the best of our knowledge there has been no such procedure performed and described so far.

**Results:**

The procedure lead to proper restoration of the defect.

**Conclusion:**

The demonstrated technique can be used to perform the reconstruction of a full-thickness osteochondral glenoid defect.

**Electronic supplementary material:**

The online version of this article (10.1007/s43465-020-00190-8) contains supplementary material, which is available to authorized users.

## Electronic supplementary material

Below is the link to the electronic supplementary material.

Supplementary material 1 (MP4 184157 kb)
